# Clinical experience of strain imaging using DENSE for detecting infarcted cardiac segments

**DOI:** 10.1186/s12968-015-0155-8

**Published:** 2015-06-24

**Authors:** Johan Kihlberg, Henrik Haraldsson, Andreas Sigfridsson, Tino Ebbers, Jan E. Engvall

**Affiliations:** Department of Radiology and Department of Medical and Health Sciences, Linköping University, Linköping, Sweden; Center for Medical Image Science and Visualization (CMIV), Linköping University, Linköping, Sweden; Department of Medical and Health Sciences, Linköping University, Linköping, Sweden; Department of Radiology and Biomedical Imaging, University of California, San Francisco, CA USA; Department of Clinical Physiology, Karolinska Institutet and Karolinska University Hospital, Stockholm, Sweden; Department of Clinical Physiology and Department of Medical and Health Sciences, Linköping University, Linköping, Sweden

## Abstract

**Background:**

We hypothesised that myocardial deformation determined with magnetic resonance imaging (MRI) will detect myocardial scar.

**Methods:**

Displacement Encoding with Stimulated Echoes (DENSE) was used to calculate left ventricular strain in 125 patients (29 women and 96 men) with suspected coronary artery disease. The patients also underwent cine imaging and late gadolinium enhancement. 57 patients had a scar area >1 % in at least one segment, 23 were considered free from coronary artery disease (control group) and 45 had pathological findings but no scar (mixed group). Peak strain was calculated in eight combinations: radial and circumferential strain in transmural, subendocardial and epicardial layers derived from short axis acquisition, and transmural longitudinal and radial strain derived from long axis acquisitions. In addition, the difference between strain in affected segments and reference segments, “differential strain”, from the control group was analysed.

**Results:**

In receiver-operator-characteristic analysis for the detection of 50 % transmurality, circumferential strain performed best with area-under-curve (AUC) of 0.94. Using a cut-off value of -17 %, sensitivity was 95 % at a specificity of 80 %. AUC did not further improve with differential strain. There were significant differences between the control group and global strain circumferential direction (-17 % versus -12 %) and in the longitudinal direction (-13 % versus -10 %). Interobserver and scan-rescan reproducibility was high with an intraclass correlation coefficient (ICC) >0.93.

**Conclusions:**

DENSE-derived circumferential strain may be used for the detection of myocardial segments with >50 % scar area. The repeatability of strain is satisfactory. DENSE-derived global strain agrees with other global measures of left ventricular ejection fraction.

## Background

Deformation abnormalities of the left ventricular myocardium may have many causes e.g., myocardial scar, ischemia or electrical conduction delay. Abnormal deformation has been objectively identified by both cardiovascular magnetic resonance (CMR) and echocardiography. Echocardiography has become the standard imaging technique for the heart in clinical routine, and offers quantitative measures of velocity and strain of the myocardium by both Doppler imaging and speckle tracking. However, CMR has become an important complement by offering superior tissue contrast and signal-to-noise ratio [1]. Strain analysis by CMR is currently not routinely recommended in practice guidelines [2], but many studies indicate that global strain has potential to uncover early systolic changes not picked up by ejection fraction alone [3]. Cardiac computerized tomography is useful for visualizing the coronary arteries but for time-resolved acquisition of ventricular function, the absorbed radiation dose sets the limit [1]. Myocardial tagging CMR [4] has been the gold standard in deformation imaging, but suffers from two main drawbacks: the analysis is time consuming and the tag lines fade over time which reduces the accuracy of the analysis [1]. Competing techniques such as Displacement Encoding with Stimulated Echoes (DENSE) [5] and strain encoding (SENC) [6] imaging have emerged and show some promising results.

Using DENSE CMR, the displacement information is encoded into the phase of each voxel in the image. This technique can be applied in 2D [5, 7–9] as well as in 3D [10]. The output of the method is a displacement map that can be used for calculating strain in various layers and directions. The theoretical advantage over tagging is the direct determination of displacement and a higher spatial resolution [11]. DENSE has been validated in phantoms [12, 13] and has been tried for detecting myocardial scar [9, 14–16] but there is a need for studies with larger patient numbers expressing different levels of myocardial dysfunction. The aim of this study was to determine the sensitivity and specificity of DENSE in detecting myocardial scar in patients with a high likelihood of coronary heart disease.

## Methods

### Patient population

One hundred and twenty five patients participating in the multicentre study “Doppler-CIP” [17] at Linköping University Hospital were included between November 2010 and March 2012. The patients were on the waiting list for myocardial scintigraphy or had a positive exercise test and a high likelihood of having coronary heart disease. Initial exclusion criteria were unwillingness to participate or those related to performing CMR such as pacemaker, atrial fibrillation, claustrophobia or a calculated glomerular filtration rate <60 ml/m^2^ [18–20]. All patients underwent contrast enhanced CMR and had blood pressure recorded at rest within 2 h of the MR exam.

The study complied with the Declaration of Helsinki and with agreements on Good Clinical Practice. Approval was obtained from the Regional Ethical Review Board in Linköping. Written informed consent was obtained from all study participants.

### CMR

CMR was performed on a Philips 1.5 T Achieva Nova Dual scanner (Philips Medical Systems, Best, the Netherlands) using a five-element cardiac synergy surface coil. All patients underwent cine CMR for visual examination, DENSE to assess cardiac function, and late gadolinium enhancement (LGE) to determine regional viability. All acquisitions were performed using breath-hold regime. The cine-CMR was performed with a balanced steady state free precession (bSSFP) sequence covering the left ventricle from apex to base in the short axis (SA) orientation as well as three long axis (LA) planes (2- and 4- chamber views as well as the apical LA view). Temporal resolution ranged between 24–41 ms, mean 31 ms (30 acquired phases).

DENSE imaging was performed in three SA slices equally spaced between apex and base as well as in three LA slices. Three targeted time frames were acquired: at the closure of the aortic valve and 45 ms before and after valve closure, in order to obtain high SNR of the DENSE acquisition [21]. Measurements obtained at the aortic valve closure time point were used in the analysis. Three in-plane displacement encoding directions were recorded using balanced multipoint encoding [22], and each direction was measured using three-fold spatial modulation of magnetization (3-SPAMM) [23]. An in-plane displacement encoding strength of 0.30 Hz/pixel was used, and through-slice dephasing of 0.25 Hz was used. In order to suppress fat signal, the first RF pulse in the DENSE 1–1 SPAMM encoding block was modified to be water-selective only. K-space was traversed with six spiral interleaves of 8 ms, TR/TE 1.27/11.2 ms [24]. Three interleaves were recorded per time frame and cardiac cycle, resulting in a total acquisition time of 18 heart beats per slice. The flip angles were chosen to optimize for maximum constant SNR for all excitations [25]. The spatial resolution was 2.73 × 2.73 mm with a reconstructed resolution of 1.36 × 1.36 mm and slice thickness 6 mm.

After DENSE imaging, LGE was acquired in the same position as the cine slices, about 20 min after the administration of gadopentetate dimeglumine (Gd-DTPA) 0.2 mmol/kg bodyweight (Bayer Healthcare, Berlin, Germany). The inversion recovery turbo field echo (IR-TFE) sequence was a segmented 3D spoiled gradient echo sequence with TE = 1.3 ms, TR = 4.4 ms and a fast spoiled gradient echo factor of 43. Slice thickness was 10 mm with a slice gap of −5 mm.

### Image analysis

The left ventricle was divided into 16 segments [26] excluding the apical cap. Patients without signs of pathology (i.e., no signs of scar, normal in terms of wall motion, blood pressure, ejection fraction and myocardial mass) comprised the control group. All images were anonymized, and the observers were blinded for other magnetic resonance images acquired at the time of the investigation.

### Left ventricular size, mass and function

Left ventricular end-diastolic and end-systolic volumes, left ventricular mass and ejection fraction were all determined from cine SA slices [27] by an experienced observer using Extended Workstation R3.2, Philips Medical Systems, Best, the Netherlands. Wall motion was visually determined using a qualitative scoring system used in echocardiography, where 1 = normal, 2 = hypokinetic, 3 = akinetic and 4 = dyskinetic [28].

### Infarct size and transmurality

A scar segment was defined as any segment with an LGE positive area exceeding one percent, with an ischemic pattern, i.e., mainly subendocardial distribution. Myocardial scar was segmented from a stack of short axis LGE images by one experienced observer, using the software “Segment v 1.9 R2966”, (http://segment.heiberg.se) [29]. A scar region was defined based on an increase in signal intensity with manual correction as needed [30]. Scar segments were divided into four groups according to the transmurality of scar expressed as scar area per segment, (1–25 %, 26–50 %, 51–75 % and >75 % transmurality). The four groups were also reduced to two based on scar area less than or in excess of 50 %.

### DENSE analysis

Two observers analysed strain in the radial and circumferential directions from the three SA slices and in the longitudinal and radial directions from the three LA slices. Lagrangian strain was reported as subendocardial, epicardial and transmural, defining “subendocardial” as 50 % of wall thickness starting from the endocardial surface, resulting in eight combinations of strain layers and directions. Differential strain was defined as the mean segmental strain value in the control group subtracted with the measured value in this particular position of a patient. All post processing was performed in Matlab (R2010b, Mathworks, Natick, MA, U.S.A.) using an in-house developed software. Interobserver variability was analysed in ten patients and the effect of repeated acquisitions was analysed in nine patients who were scanned twice for DENSE slices in the SA direction without being released from the scanning table between the two acquisitions.

### Statistical analysis

Statistical analysis was performed using SPSS 20 (SPSS Inc., Chicago, Illinois, USA). All variables were reasonably normally distributed why parametric tests were used. For multiple comparisons, analysis of variance with Bonferroni correction was used. Inter-observer and scan-rescan variability of the functional measures was expressed as intra class correlation coefficient (ICC). All statistical testing used a significance level of <0.05. Receiver operator curves (ROC) were calculated for all strain values with late gadolinium enhancement transmurality >50 % as end point.

## Results

### Study population

In total 125 patients were included in this study (mean age 67 years, range 49–85 years, 96 males). 48 patients had a self-reported history of previous myocardial infarction (MI), 41 patients had undergone PCI and 10 had received Coronary Artery Bypass Graft (CABG) surgery. 14 patients had had a myocardial infarction within 1 year, average time delay 179 days.

Out of the 125 patients, 57 patients had at least one segment with positive LGE of more than one percent transmurality (46 %), 34 had a transmurality >50 % (27 %) while 23 showed very low probability of coronary artery disease (control group, 18 %) based on normal wall motion, blood pressure (BP), LVEF, LVM and absence of signs of scar and self-reported prolonged chest pain. 45 patients had one or more pathological findings but less than 1 % scar (mixed group, 32 %). There were no significant differences in age, gender or body mass index (BMI) between the groups. Nine patients had not experienced chest pain and were presumed to have had a silent MI.

### Left ventricular volumes, LVEF and blood pressure

Left ventricular end-diastolic volume (LVEDV) and end-systolic volume (LVESV) were significantly larger and left ventricular ejection fraction (LVEF) lower in the scar group compared with the control group, as expected (Table 1). Systolic blood pressure did not differ between the three groups.

**Table 1:** Left ventricular volumes, left ventricular mass and left ventricular scar volume

	Scar > 1 %	*p*-value	Control	*p*-value	Mixed	*p*-value
(*N* = 125)	(*n* = 57)	LGE-Ctrl	(*n* = 23)	Ctrl-Mix	(*n* = 45)	Mix-LGE
LVEDV, mL	168 ± 40	0.076	148 ± 26	NS	145 ± 36	0.007
LVESV, mL	82 ± 38	<0.001	50 ± 15	NS	54 ± 23	<0.001
LVEF, %	52 ± 13	<0.001	66 ± 6	NS	62 ± 9	<0.001
LVmass, g	60 ± 13	0.073	52 ± 7	NS	56 ± 14	0.345
LVscar, %	9 ± 8	N/A	N/A	N/A	0.6 ± 2	<0.001

Left ventricular end-diastolic (LVESV) and end-systolic volumes (LVESV) (mL), left ventricular ejection fraction (LVEF), left ventricular mass corrected for body surface area (LVmass) (gram) and the scar volume fraction of the left ventricle (%). Mean value ± standard deviation, ANOVA with Bonferroni post hoc test, N/A = Not Applicable. Table 2 - Global strain values for patients with any segment scar > 50 % and the no scar control group

Patients with a recent MI compared with those that had experienced an infarct more than one year earlier showed no significant difference in LVEDV, LVESV, LVM, LVEF or the mean global value of strain in the radial, circumferential and longitudinal directions.

### Segmental scar area and LV mass

Scar (LGE > 1 %) was present in 57 patients and in 385 segments (19 % of total number of segments). The number of segments with scar area 1–25 % was 202, 26–50 % was 95, 51–75 % was 60 and >75 % was 28. Eighty-eight segments in 34 patients had more than 50 % transmurality. Scar size was on average 12 ± 12 ml or 9.3 ± 8.1 % of the left ventricular myocardium. LVM was significantly larger in the scar group compared to both the control and the mixed group (Table 1). A wall motion abnormality was deemed present in 252 segments affecting 56 patients.

### Strain measurements

**Fig. 1:**
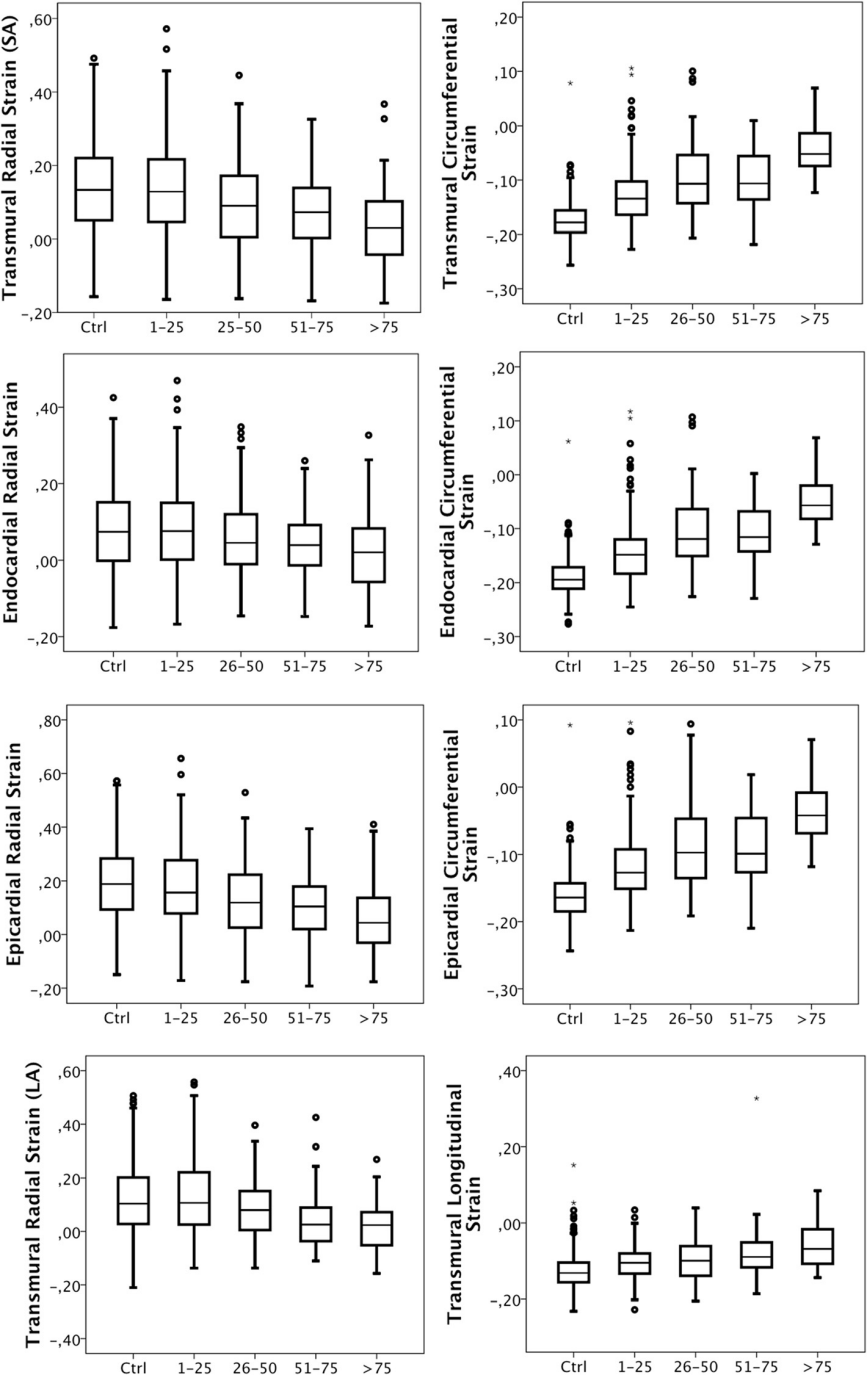
Layer strain versus segmental transmurality of scar. The box plots show median, the two central quartiles in the box, one quartile in each wisker and outliers. The upper three rows show radial (*left*) and circumferential (*right*) strain boxplots obtained from transmural (*top*), subendocardial (2nd from top) and epicardial (*3rd from top*) measurements. The fourth row shows transmural radial (*left*) and lonitudinal (*right*) strain obtained from the long axis in segments with various degree of transmurality of scar

Strain was determined with DENSE in 16 segments, for a combination of 8 layers and directions creating 128 measurements per patient. The success rate was 94 % (14 986 of 16 000 measurements). Failure to determined strain was in all cases due to inability of the patients to hold breath for approximately 15 s. Results from the DENSE analysis are reported in Fig. 1. Strain from the SA acquisition is given in the circumferential and radial direction and divided into transmural, subendocardial and epicardial components (Fig. 1, upper 6 panels). Subendocardial strain was on average higher than epicardial and transmural strain for all patients. Strain from the LA slices was calculated as longitudinal transmural and radial transmural strain (Fig. 1, lowest panels). For incremental increases in transmurality of scar, the absolute value of strain was reduced in all directions and layers (Fig. 1).

**Fig. 2:**
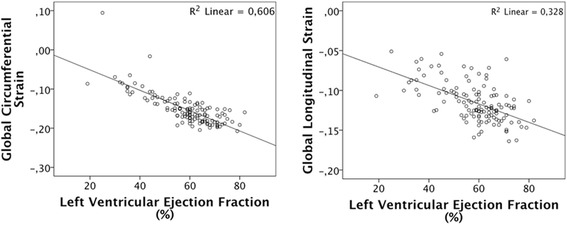
Global circumferential and longitudinal strain as functions of left ventricular ejection fraction. Global strain (average of 16 segments of the left ventricle) versus left ventricular ejection fraction. Circumferential strain to the left, longitudinal strain to the right

When analysed as global strain (mean of 16 segments per patient), scar patients had lower absolute values than the control group (Table 2). The correlation between global circumferential strain and LVEF was high. The correlation was lower but still significant for global longitudinal strain versus LVEF (Fig. 2).

**Table 2:** Transmural global strain for patients with LGE more than 50 % and control patients is presented as mean value ± standard deviation, together with its p-value

Strain direction	LGE > 50 %	Control	*p*-value
(*N* = 57)	(*n* = 34)	(*n* = 23)	
Radial transmural strain (SA)	12 ± 6	14 ± 6	0.12
Radial transmural strain (LA)	10 ± 6	11 ± 7	0.65
Circumferential transmural strain	−12 ± 4	−17 ± 5	<0.001
Longitudinal transmural strain	−10 ± 2	−13 ± 2	<0.001

Transmural global strain expressed as percentage with 1 SD

In the control group strain increased from the apex to the base for the radial component, but in the longitudinal and circumferential directions, strain was more uniform. The standard deviation was smaller in circumferential and longitudinal strain than in radial strain (Table 3). There were no significant differences in strain between the mixed group and the scar group or the control group.

**Table 3:** Segmental distribution of strain values of the left ventricle in the control group

Type of strain		Anterior	Anteroseptal	Inferoseptal	Inferior	Inferolateral	Anterolateral
Radial transmural strain SA							
	Basal	20 ± 8	15 ± 9	16 ± 8	21 ± 9	28 ± 13	29 ± 10
	Mid-ventricular	13 ± 7	10 ± 7	12 ± 7	13 ± 8	23 ± 10	22 ± 11
	Apical	1 ± 7	−4 ± 6	-	2 ± 9	-	8 ± 9
Radial transmural strain LA							
	Basal	18 ± 11	7 ± 11	12 ± 7	22 ± 10	22 ± 13	24 ± 14
	Mid-ventricular	22 ± 13	8 ± 10	8 ± 7	12 ± 8	12 ± 18	16 ± 12
	Apical	2 ± 11	2 ± 8	-	6 ± 11	-	−1 ± 10
Radial subendocardial strain							
	Basal	12 ± 7	8 ± 8	10 ± 6	15 ± 9	21 ± 11	21 ± 9
	Mid-ventricular	7 ± 6	4 ± 6	5 ± 6	7 ± 7	17 ± 9	15 ± 10
	Apical	−5 ± 6	−8 ± 5	-	−2 ± 7	-	2 ± 8
Radial epicardial strain							
	Basal	26 ± 9	22 ± 11	21 ± 9	26 ± 10	33 ± 14	35 ± 11
	Mid-ventricular	17 ± 8	15 ± 8	18 ± 8	18 ± 10	29 ± 12	28 ± 12
	Apical	5 ± 8	−1 ± 8	-	6 ± 11	-	12 ± 11
Circumferential transmural strain							
	Basal	−16 ± 3	−14 ± 4	−14 ± 3	−15 ± 3	−20 ± 2	−18 ± 3
	Mid-ventricular	−19 ± 2	−18 ± 3	−15 ± 3	−18 ± 3	−19 ± 2	−18 ± 2
	Apical	−20 ± 3	−19 ± 3	-	−17 ± 6	-	−19 ± 4
Circumferential subendocardial strain							
	Basal	−18 ± 3	−17 ± 4	−16 ± 3	−17 ± 3	−21 ± 2	−19 ± 3
	Mid-ventricular	−21 ± 2	−20 ± 2	−17 ± 2	−19 ± 3	−20 ± 2	−20 ± 2
	Apical	−21 ± 3	−20 ± 3	-	−19 ± 6	-	−20 ± 4
Circumferential epicardial strain							
	Basal	−15 ± 3	−13 ± 4	−13 ± 3	−14 ± 4	−18 ± 2	−17 ± 3
	Mid-ventricular	−18 ± 2	−17 ± 3	−13 ± 3	−16 ± 3	−17 ± 2	−17 ± 2
	Apical	−19 ± 2	−18 ± 3	-	−16 ± 6	-	−18 ± 4
Longitudinal transmural strain							
	Basal	−11 ± 6	−10 ± 4	−12 ± 3	−14 ± 5	−17 ± 4	−17 ± 3
	Mid-ventricular	−11 ± 3	−14 ± 3	−15 ± 2	−15 ± 3	−15 ± 3	−14 ± 3
	Apical	−6 ± 5	−13 ± 2	-	−13 ± 5	-	−10 ± 3

Average segmental strain values for the control group excluding the apical cap. Segmentation of the left ventricle according to the American Heart Association. Strain values at the base, mid and apical levels are all significantly different from each other (ANOVA with Bonferroni post hoc test, *p* < 0.007)

### Sensitivity and specificity

**Fig. 3:**
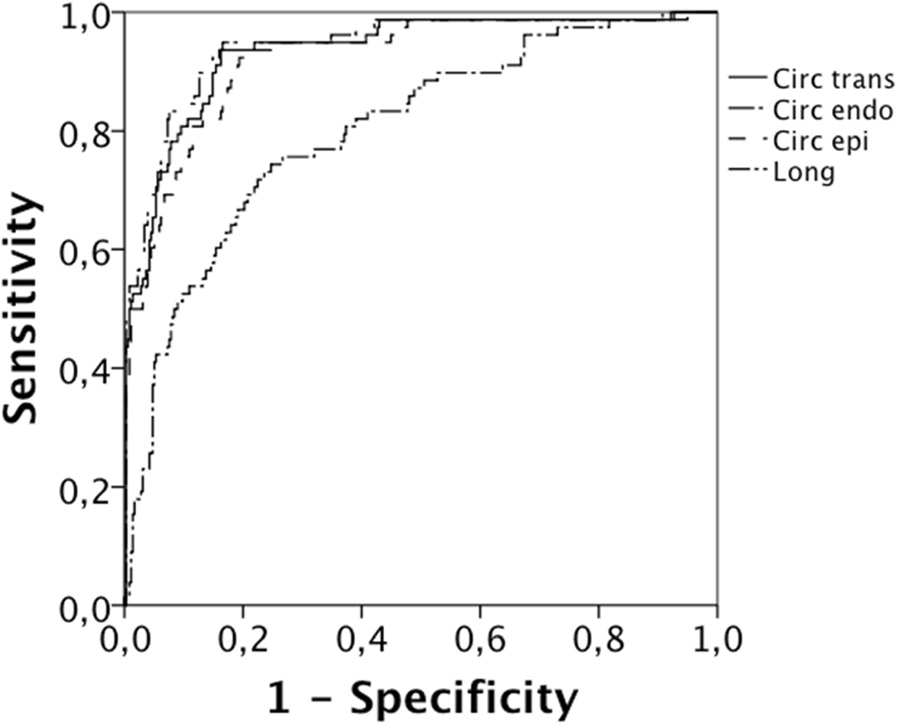
ROC curves for layer strain and the detection of >50 % transmurality of scar. Circ trans = transmural circumferential strain, Circ endo = subendocardial circumferential strain, Circ epi = epicardial circumferential strain and Long = Longitudinal strain

Receiver-operator curves were constructed for different combinations of strain directions and layers to assess their ability to identify segments with transmurality >50 %. Fig. 3 shows Area-under-curve (AUC) in excess of 0.80 for the eight combinations of strain layers and directions, which are four combinations; circumferential subendocardial, circumferential epicardial, circumferential transmural and longitudinal transmural strain.

**Fig. 4:**
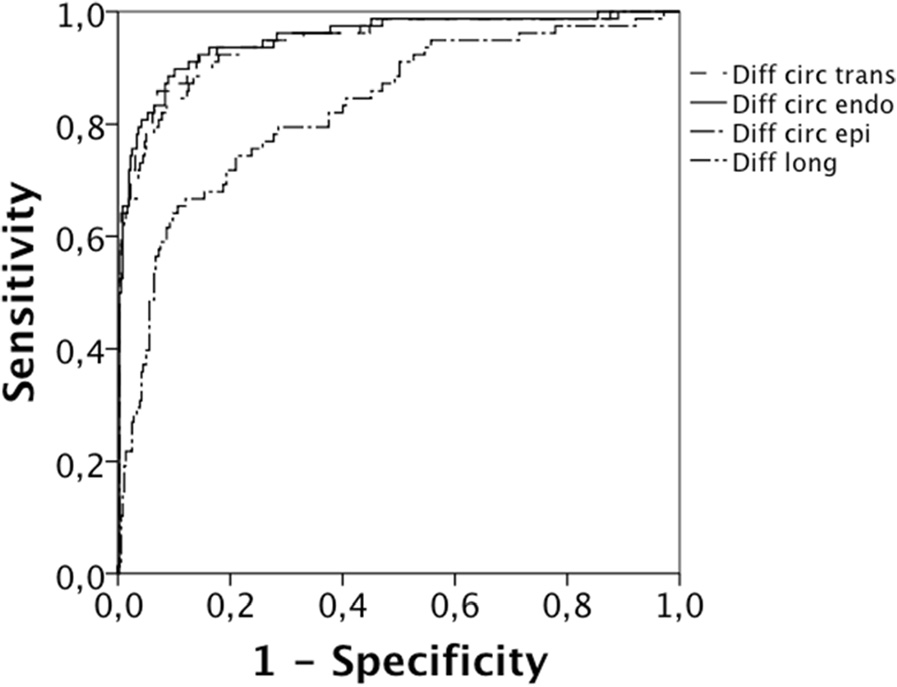
ROC curves for layer strain expressed as differential strain versus the detection of >50 % transmurality of scar. Diff circ endo = subendocardial circumferential differential strain, Diff circ epi = epicardial circumferential differential strain, Diff circ = transmural circumferential differential strain, Diff long = longitudinal differential strain

AUC for various strain components is shown in Table 4, which also depicts sensitivity and specificity for different cut-off levels. Best AUC was for subendocardial circumferential strain, which detected segments with scar area >50 % with 94 % sensitivity at 80 % specificity. Analysis of differential strain (Fig. 4, Table 5) did not significantly improve the detection rate for scar.

**Table 4:** AUC for direction and layer of strain versus >50 % transmurality of scar

Direction and layer of strain	AUC	Sens (%) at spec = 80 %	Strain cut-off value	Scar	Control	*p*-value
Radial subendocardial strain SA	0.60	27	N/A	4 ± 10	9 ± 11	0.002
Radial epicardial strain SA	0.69	45	N/A	9 ± 13	19 ± 14	<0.001
Radial transmural strain SA	0.66	39	N/A	7 ± 11	14 ± 13	<0.001
Radial transmural radial strain LA	0.67	40	N/A	4 ± 11	12 ± 13	<0.001
Circumferential subendocardial strain	0.94	95	−17	−9 ± 6	−19 ± 3	<0.001
Circumferential epicardial strain	0.92	91	−14	−7 ± 5	−16 ± 4	<0.001
Circumferential transmural strain	0.93	94	−15	−8 ± 5	−17 ± 4	<0.001
Longitudinal transmural strain	0.80	66	−10	−7 ± 7	−12 ± 4	<0.001

Strain for segments with transmurality in excess of 50 % (80 segments) is compared with strain in 356 control segments, regardless of location. AUC is presented together with sensitivity at 80 % specificity and the corresponding cut-off value for strain, when applicable. For strain with AUC less than 0.70, a cut-off value was not calculated, N/A = Not Applicable

**Table 5:** ROC analysis for differential strain as shown in Fig. 3

Type and layer of strain	AUC	Sens (%) at spec = 80 %	Diff strain cut-off value
Radial subendocardial strain SA	0.51	18	N/A
Radial epicardial strain SA	0.66	36	N/A
Radial transmural strain SA	0.61	30	N/A
Radial transmural radial strain LA	0.63	28	N/A
Circumferential subendocardial strain	0.95	94	−2
Circumferential epicardial strain	0.94	91	−2
Circumferential transmural strain	0.95	94	−2
Longitudinal transmural strain	0.83	72	−2

Differential strain (for definition see text) for segments with transmurality in excess of 50 % (80 segments) is compared with differential strain in 356 control segments, regardless of location. AUC is presented together with sensitivity at 80 % specificity and the corresponding cut-off value for differential strain, when available

### Inter-observer and scan-rescan variability

Table 6 shows that reproducibility was excellent with an Intra Class Correlation Coefficient (ICC) in excess of 0.93 for all measured directions for inter-observer variability and 0.95 for scan-rescan variability.

**Table 6:** Interobserver and scan-rescan variability

	Inter observer		Scan-rescan	
	ICC	95 % CI	ICC	95 % CI
Radial transmural strain SA	0.96	0.95–0.97	0.95	0.92–0.96
Radial transmural strain LA	0.93	0.91–0.95	-	-
Circumferential transmural strain	0.99	0.98–0.99	0.96	0.94–0.97
Longitudinal transmural strain	0.96	0.94–0.97	-	-

Inter-observer variability (two observers measuring on the same images) in 10 patients and scan-rescan results from one observer in 9 patients in the short axis direction. Intraclass Correlation Coefficient (ICC) is shown with 95 % Confidence Interval (CI)

## Discussion

In this study, we evaluated the ability of DENSE CMR based assessment of myocardial deformation to detect myocardial scar. Peak strain was calculated in the radial, longitudinal and circumferential directions derived from the transmural, subendocardial and epicardial layers. We found that circumferential strain detects segments with scar transmurality >50 % with high sensitivity and specificity and excellent inter-observer variability and a low scan-rescan variability. Radial and longitudinal strain showed excellent and inter-observer and scan-rescan variability, but lower sensitivity and specificity for the detection of scar. In the study of Miyagi et al., circumferential strain was expressed in technical terms as “E2” and displayed the best diagnostic performance for detecting myocardial scar compared with other expressions of strain [16]. Furthermore, our result based on circumferential strain was better than what has been previously shown with feature tracking [31].

Subendocardial circumferential strain had slightly higher sensitivity than epicardial and transmural strain in detecting scar (Table 4). This is to be expected since infarct scar most often has its largest extent in the subendocardial layer. We also found that global strain decreases with increasing scar size. This can be explained by low strain in scar areas and also low strain in dilated remodelled ventricles such as can be seen after a myocardial infarction.

The temporal development of LV remodelling after a myocardial infarction has been monitored by CMR strain in a previous study [32]. Contrary to that study, the time elapsed after the infarct in our study did not seem to influence either global strain or LV volume. However, the patients included in our study had few recent infarcts (within one year) and the elapsed time was quite long (6 months on average).

Radial strain acquired with DENSE has previously been reported to display greater variability than circumferential strain. Our results also show a greater standard deviation in radial strain in the control subjects and low AUC for detecting scar. The absolute values seen here are considerably lower than radial strain values obtained with other techniques such as feature tracking [31]. Comparably low values have been shown with DENSE in previously healthy persons [33]. This could be related to the different measurement techniques used. In feature tracking ultrasound and in tagging CMR, the radial strain estimate heavily depends on how well the myocardial border is detected, while in DENSE CMR only the encoded displacement of myocardial tissue is used. The presence of trabeculae and the relatively thin myocardium might affect these techniques differently. Another contributing factor might be regional variation in radial strain in the controls. Even the application of differential strain which subtracts the measured value from a normal value obtained separately for location (base-mid-apical areas of the heart), AUC was lower for radial strain compared with circumferential strain in all layers. Radial strain derived from the LA acquisition did not differ from SA radial strain. Using global radial strain, hearts with large scar could not be differentiated from those without scar because of the large standard deviation. This supports previous reports that DENSE, as currently applied, has a greater variation in the radial strain component [34].

The reproducibility of myocardial strain acquired with DENSE was very good, despite a short time allocated for practising segmentation. In addition, a second scan evaluating the test-retest situation, gave values for interobserver variability in the same range as those obtained for the test-retest situation. This shows the robustness of strain acquisition using DENSE. The patient cohort was recruited from the waiting list for myocardial scintigraphy with a high calculated pre-test probability of coronary heart disease. However, some of the patients were normal from the point of view of atherosclerotic coronary artery disease, as far as can be ascertained without resorting to angiography. Thus we believe that the claim that the reference group was “cardiovascularly healthy” is valid. Furthermore, the age and gender distribution is similar to that of the clinical patient group.

Strain can be expressed in reference to Lagrangian or Eulerian coordinates [35] as well as engineering strain [36]. Eulerian strain will have a lower value in positive strain and Lagrangian lower absolute value in negative strain. Unfortunately, there is no consensus on which frame of reference to use when reporting myocardial strain [37]. We selected Lagrangian strain since it is frequently used in biomechanics.

### Limitations

In the definition of the “scar”, “mixed” and “healthy” groups, “healthy” patients fulfilled the general inclusion criteria but were found to have a low post-test likelihood of disease after completed investigations and were re-classified as “healthy”. In the calculations, the high spatial resolution of DENSE was partly lost since values were averaged over the entire LV. Nearby scar segments may influence segmental strain values, but this effect could be minimized by the high spatial resolution of DENSE. Strain was determined at the closure of the aortic valve which may have caused the true peak to be missed. Repeated analysis over the entire cardiac cycle (“cine DENSE”) could perhaps provide greater temporal resolution.

## Conclusions

DENSE CMR is able to detect progressively increasing segmental scar area. Cut-off values for circumferential strain detected a segment with scar area >50 % with 94 % sensitivity at 80 % specificity. The repeatability of the circumferential measurements of strain is excellent and global strain agrees with other aspects of global left ventricular systolic function such as LVEF. This indicates that DENSE can be a clinically useful tool for measuring myocardial strain.

## Abbreviations

ANOVA: Analysis of variance
CABG: Coronary artery bypass graft
CMR: Cardiovascular magnetic resonance
DENSE: Displacement encoding with stimulated echoes
ECG: ElectroCardioGram
ICC: Intraclass correlation coefficient
IR-TFE: Inversion recovery turbo field echo
LA: Long axis
LGE: Late gadolinium enhancement
LV: Left ventricle
LVEF: Left ventricular ejection fraction
LVEDV: Left ventricular end-diastolic volume
LVESV: Left ventricular end-systolic volume
LVM: Left ventricular mass
MR: Magnetic resonance
PCI: Percutaneous coronary intervention
ROC: Receiver-operator-characteristics
SA: Short axis
SD: Standard deviation
SSFP: Steady state free precession
TE: Echo time
TR: Repetition time
